# From Expected Goals to Scoring at Least Once: An Event-Specific Summary of Aggregated Bernoulli Risk

**DOI:** 10.3390/e28050527

**Published:** 2026-05-06

**Authors:** Tomasz Górecki

**Affiliations:** Faculty of Mathematics and Computer Science, Adam Mickiewicz University, 61-614 Poznań, Poland; tomasz.gorecki@amu.edu.pl

**Keywords:** expected goals, scoring certainty, shot concentration, Poisson–binomial model, effective number of shots, Rényi entropy

## Abstract

Expected goals (xG) is widely used to quantify offensive performance in football by summarizing the expected number of goals from shot-level scoring probabilities. However, xG reflects only the first moment of the underlying Bernoulli system and does not capture how scoring probability is distributed across shots. As a result, teams with identical total xG may nevertheless have different probabilities of scoring at least once. In this paper, we study the quantity $xG^{+}=-\log P\left(G=0\right)$, which is a monotone transform of the exact no-goal probability and, equivalently, of the probability of scoring at least once. We interpret $xG^{+}$ as an additive, event-specific summary of aggregated Bernoulli risk and analyze its main structural properties. In particular, we show that $xG^{+}\geq xG$, with equality only in the degenerate case $p_{i}=0$ for all *i*, and we derive a second-order approximation linking $xG^{+}-xG$ to the second moment of shot probabilities, the effective number of shots, and Rényi-2 entropy. Empirical illustrations on football data show how concentrated shot profiles can increase scoring certainty relative to total xG and how exact Bernoulli aggregation differs from a Poisson approximation based only on the mean. While xG remains an appropriate measure of expected scoring volume, $xG^{+}$ provides a complementary summary targeted at the probability of scoring at least once.

## 1. Introduction

Expected goals (xG) has become a standard summary of offensive production in football. At the shot level, an xG model assigns a scoring probability to each attempt using spatial, contextual, and event-related information; at the match level, total xG is obtained by summing these shot-level probabilities. This construction is intuitive, operationally useful, and easily interpretable because total xG directly estimates the expected number of goals a team generates [1,2,3]. Consequently, xG has become a central quantity in performance analysis, recruitment, tactical review, and forecasting.

From a probabilistic perspective, shot outcomes correspond to a sequence of Bernoulli trials whose sum follows a Poisson–binomial distribution. In practice, aggregated expected goals are often approximated using a Poisson distribution with rate parameter equal to total xG [4,5]. This approximation is natural when the target is expected scoring volume because it matches the mean of the shot-level Bernoulli sum. However, it does not capture how the distribution of shot probabilities affects event-level outcomes.

Yet total xG is only a first-moment descriptor of a heterogeneous Bernoulli system. If a team produces shot-level probabilities $p_{1},\ldots ,p_{n}$, then

(1) $$xG=\sum_{i=1}^{n}p_{i}.$$

This aggregation preserves the mean but discards information about how scoring probability is distributed across shots. In football, this loss of structure is consequential. Two shot profiles with identical total xG may imply substantially different probabilities of scoring at least one goal and, more generally, different distributions of the number of goals scored.

This places expected goals analysis within the broader class of aggregated Bernoulli systems, where first moments do not fully characterize probabilistic behavior [6]. In such settings, the distribution of probability mass across components becomes important. In the present framework, the exact analytical structure is governed by the scoreless-match probability and, at the approximation level, by the second moment of shot probabilities. The effective number of shots and Rényi entropy of order two provide convenient normalized reformulations of this concentration structure, whereas Shannon entropy [7] plays only a secondary descriptive role.

Although information-theoretic ideas have appeared in football analytics, they have primarily been used to study league dynamics, passing organization, or event unpredictability rather than shot-level scoring certainty [8,9,10,11]. In parallel, the expected goals literature has focused on estimating shot-level scoring probabilities and on practical applications of xG [1,2,12,13]. The present work connects these strands by focusing on certainty about scoring at least once as a functional summary of aggregated Bernoulli risk.

Our point of departure is simple: if the objective is expected scoring volume, then xG is the natural summary; if the objective is certainty about scoring at least once, then the mean alone is not sufficient. To capture this aspect, we consider the quantity

(2) $$xG^{+}=-\log P\left(G=0\right),$$

where *G* denotes the total number of goals and P(G=0) is the exact scoreless-match probability in the shot-level Bernoulli system. Thus, $xG^{+}$ is event-specific: it targets certainty about G≥1 rather than expected goal volume. Equivalently, it is the value of the Poisson parameter that reproduces the same probability of a scoreless outcome as the underlying shot-level Bernoulli system, that is, the unique λ such that $P_{\mathrm{Pois}(\lambda )}\left(G=0\right)=P\left(G=0\right)$.

The quantity $xG^{+}$ is a monotone transformation of the exact no-goal probability P(G=0) and, equivalently, of the exact probability of scoring at least once, P(G≥1). Consequently, larger values of $xG^{+}$ correspond exactly to greater scoring certainty. At the same time, unlike the probability scale itself, $xG^{+}$ admits an additive representation across shots:

$$xG^{+}\left(p\right)=\sum_{i=1}^{n}-\log\left(1-p_{i}\right).$$

This additive form makes it analytically convenient for studying how heterogeneity in shot-level probabilities affects scoring certainty.

The mapping from the shot-level probability vector $p=(p_{1},\ldots ,p_{n})$ to $xG^{+}\left(p\right)$ is nonlinear and convex in each component. These structural properties underlie the dominance of $xG^{+}$ over xG, its sensitivity to concentration, and the Jensen- and majorization-based results developed below. In particular, $xG^{+}$ always exceeds or equals total xG, and the discrepancy $xG^{+}-xG$ is driven by concentration in shot-level probabilities. This links scoring certainty to the second moment, the effective number of shots $N_{\mathrm{eff}}$, and Rényi entropy of order two.

The main contribution of this paper is not the mere observation that $xG^{+}$ is a monotone transformation of the exact probability of scoring at least once. Rather, the contribution is to identify and interpret $xG^{+}$ as an additive, event-specific summary of aggregated Bernoulli risk and to study its structural properties within this framework. The theoretical contribution is therefore structural and interpretive rather than primarily technical: we collect and organize the key properties of $xG^{+}$, including its dominance over xG, its dependence on shot concentration, and its connection to second-moment effects and Poisson-induced approximations. The empirical analysis is included as an illustration of these ideas in football data, rather than as the primary source of novelty. Throughout, the theoretical development is conditional on the standard independence approximation at the shot level, which serves here as a transparent reference model rather than a full description of match dynamics.

**Figure 1:**
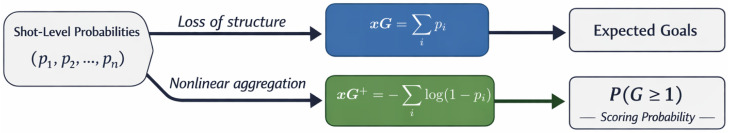
Conceptual illustration of two additive summaries derived from the same shot-level probability system $\left(p_{1},\ldots ,p_{n}\right)$. The standard expected goals measure $xG=\sum p_{i}$ captures expected scoring volume but ignores the distribution of probabilities across shots. In contrast, $xG^{+}=-\sum \log\left(1-p_{i}\right)$ applies a nonlinear transformation that preserves additivity while encoding the probability of scoring at least one goal. The upper path represents a lossy aggregation of the underlying Bernoulli structure, whereas the lower path retains information relevant to scoring certainty.

This distinction is illustrated schematically in Figure 1. The same shot-level probability system induces two additive summaries: xG, which captures expected scoring volume, and $xG^{+}$, which encodes scoring certainty through the probability of at least one goal.

### Motivating Example

**Figure 2:**
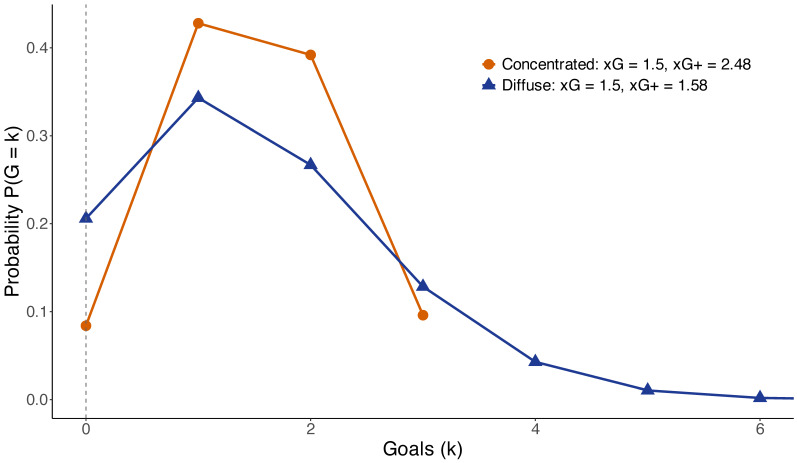
Distribution of the number of goals for two shot profiles with identical total xG=1.5 but different concentrations of shot probabilities. The concentrated profile yields a higher probability of scoring at least one goal and a heavier upper tail, despite having the same expected number of goals as the diffuse profile.

To illustrate the limitations of the total xG, consider two shot profiles with identical expected goals, xG=1.5, but different distributions of shot quality. The concentrated profile consists of three shots with probabilities (0.8,0.4,0.3), whereas the diffuse profile consists of fifteen shots with probability 0.1 each. Figure 2 shows the resulting distributions of the number of goals in the Poisson–binomial model. Although both profiles have the same expected scoring volume, their goal distributions differ substantially. In particular, the concentrated profile yields a lower probability of a scoreless outcome and a heavier upper tail, while the diffuse profile assigns more mass to low goal counts. This difference is reflected in the probability of a scoreless game. For the concentrated profile, P(G=0)=0.084, while for the diffuse profile, P(G=0)=0.206. Consequently, the corresponding values of $xG^{+}=-\log P\left(G=0\right)$ are $xG^{+}=2.477$ and $xG^{+}=1.580$, respectively. This example shows that total xG captures only the first moment of the underlying Bernoulli system and ignores the distribution of shot probabilities. As a consequence, it fails to reflect differences in scoring certainty. In contrast, $xG^{+}$ directly incorporates this information through the scoreless probability. The discrepancy between the two profiles is driven by differences beyond the mean, as they share the same total xG but differ in concentration and variance. This example shows that scoring certainty cannot be inferred from total xG alone. Table 1 summarizes the key quantities for both profiles. Despite identical total xG, the concentrated profile exhibits substantially higher scoring certainty, as reflected in larger $xG^{+}$ and lower P(G=0).

## 2. Materials and Methods

### 2.1. Shot-Level Probabilities and Aggregated Goals

Consider a team game with *n* shots. Let $X_{i}$ denote the binary outcome of shot *i*, where $X_{i}=1$ if the shot results in a goal and $X_{i}=0$ otherwise. Conditional on shot-level information, we model

(3) $$X_{i}∼\mathrm{Bernoulli}\left(p_{i}\right),\;i=1,\ldots ,n,$$

where $p_{i}\in \left[0,1\right)$ is the shot-level expected-goal probability. Assuming conditional independence across shots, given the shot-level probabilities $p_{i}$, the total number of goals scored by a team in a game is

(4) $$G=\sum_{i=1}^{n}X_{i}.$$

All theoretical results developed below are derived under this conditional independence assumption. In this formulation, the resulting distribution of *G* is Poisson–binomial [14]. This assumption is analytically convenient and standard in shot-based xG modeling, but it may be violated in practice. In particular, the tactical momentum, game state, possession structure, score effects, and other contextual factors may induce residual dependence between shots even after conditioning on shot-level covariates. Accordingly, the present framework should be interpreted as a conditional benchmark that isolates the effect of aggregating heterogeneous Bernoulli risks, rather than as a complete generative model of match dynamics. Its mean and variance are

(5) $$E\left(G\right)=\sum_{i=1}^{n}p_{i}=xG,$$

and

(6) $$V\mathrm{ar}\left(G\right)=\sum_{i=1}^{n}p_{i}\left(1-p_{i}\right).$$

Equation (6) already shows that variability depends on the full shot profile, not merely on the mean.

### 2.2. From xG to $xG^{+}$

The probability of a scoreless game is

(7) $$P\left(G=0\right)=\prod_{i=1}^{n}\left(1-p_{i}\right).$$

This yields the probability of scoring at least one goal,

(8) ScoreProb=P(G≥1)=1−P(G=0).

We define $xG^{+}$ by

(9) $$xG^{+}:=-\log P\left(G=0\right).$$

Accordingly, $xG^{+}$ is tailored to the binary event of scoring at least one goal and should not be interpreted as a full summary of the entire goal-count distribution.

Substituting Equation (7) into Equation (9) gives the additive representation

(10) $$xG^{+}=-\sum_{i=1}^{n}\log\left(1-p_{i}\right).$$

This identity implies that $xG^{+}$ is a shot-level additive quantity, just like xG, but it transforms probabilities nonlinearly in a way that emphasizes scoring certainty.

The terminology “Poisson-equivalent rate” is justified by the identity

(11) $$e^{-xG^{+}}=P\left(G=0\right),$$

meaning that $xG^{+}$ is the Poisson intensity that reproduces the exact scoreless-match probability of the underlying shot-level Bernoulli system. $xG^{+}$ is therefore the Poisson parameter that gives the same probability of zero goals as the original shot-level Bernoulli model. Thus, if one replaces the full heterogeneous shot profile by a single Poisson model, $xG^{+}$ is the value that preserves the probability of a scoreless match exactly. It should therefore be interpreted as an event-specific summary tied to the probability of scoring at least once, not as the expected number of goals. Note that $xG^{+}$ matches only the zero-event probability and does not reproduce higher moments of the Poisson–binomial distribution. In particular, it should not be interpreted as a replacement for the Poisson–binomial distribution of *G* but rather as a Poisson-equivalent rate tailored to the event G=0. This distinction is also important for interpretation: the Poisson parameter in a model based on xG has the usual meaning of expected scoring volume, whereas the parameter in a model based on $xG^{+}$ should be understood only as an event-targeted equivalent rate, not as an expected number of goals.

This construction also clarifies the relationship with Poisson-induced approximations. The standard Poisson model with rate λ=xG is induced by moment matching because it reproduces the mean of the Poisson–binomial goal distribution. By contrast, the rate $\lambda =xG^{+}$ is induced by zero-probability matching because it reproduces P(G=0) exactly. Thus, xG and $xG^{+}$ correspond to two different Poisson reductions of the same heterogeneous Bernoulli system, each aligned with a different inferential target.

Although $xG^{+}$ is expressed on a rate-like scale, it should not be interpreted as an expected number of goals. Its role is different: it is the Poisson-equivalent rate that reproduces the exact probability of a scoreless outcome, and therefore it targets the binary event of scoring at least one goal. Consequently, large values of $xG^{+}$ indicate that the event G≥1 is close to certainty, not that many goals are expected on average.

Like xG itself, $xG^{+}$ is only as reliable as the shot-level probabilities on which it is based. It should therefore be interpreted as a transformation of model-based scoring probabilities, not as a correction for misspecification in those probabilities.

### 2.3. Concentration and Entropy Measures

To quantify the structure of shot-level xG contributions, we define normalized weights for profiles with total xG>0:

(12) $$w_{i}=\frac{p_{i}}{\sum_{j=1}^{n}p_{j}}=\frac{p_{i}}{xG},\;i=1,\ldots ,n.$$

These weights form a probability distribution over the shot contributions to total xG. When xG=0, all shot probabilities satisfy $p_{i}=0$, so the normalized weights and the quantities based on them are treated as undefined.

We use the effective number of shots as the primary match-level descriptor of concentration for profiles with xG>0:

(13) $$N_{\mathrm{eff}}=\frac{{\left(\sum_{i=1}^{n}p_{i}\right)}^{2}}{\sum_{i=1}^{n}p_{i}^{2}}=\frac{xG^{2}}{\sum_{i=1}^{n}p_{i}^{2}}=\frac{1}{\sum_{i=1}^{n}w_{i}^{2}},$$

which corresponds to the inverse Simpson concentration index [15] of the distribution $\left\{w_{i}\right\}$ and may be interpreted as the number of equally contributing shots required to produce the observed total xG. When xG=0, this quantity is not defined through the normalized representation, and we do not assign it a value. The quantity $N_{\mathrm{eff}}$ can be interpreted as the number of equally probable scoring opportunities that would generate the same concentration of shot probabilities as the observed shot profile. As a secondary descriptive quantity, we also report Shannon entropy for profiles with xG>0,

(14) $$H=-\sum_{i=1}^{n}w_{i}\log w_{i},$$

which is likewise treated as undefined when xG=0. By contrast, the quantities xG and $xG^{+}$ remain well defined at the boundary case xG=0, for which necessarily $p_{i}=0$ for all *i* and hence $xG^{+}=0$. Shannon entropy is included only as a secondary descriptive quantity; the exact analytical results developed below are linked to the second moment and Rényi entropy of order two rather than to Shannon entropy. Entropy is high when xG is distributed relatively evenly across shots and low when xG is concentrated in a few attempts.

### 2.4. Event Probabilities

Two event probabilities are central to the empirical comparison. The first is the scoreless-match probability P(G=0). The second is the scoring probability P(G≥1)=1−P(G=0). Using a Poisson model with rate λ=xG, these become

(15) $$P_{\mathrm{Pois}(xG)}\left(G=0\right)=e^{-xG}\;\mathrm{and}\;P_{\mathrm{Pois}(xG)}\left(G\geq 1\right)=1-e^{-xG}.$$

Under $xG^{+}$, the probability of scoring at least once is

(16) $$P_{xG^{+}}\left(G\geq 1\right)=1-e^{-xG^{+}}=1-P\left(G=0\right),$$

which exactly matches the shot-level Bernoulli system. Accordingly, Pois(xG) and Pois$\left(xG^{+}\right)$ should be understood as event-targeted approximations derived from different matching principles. The former matches the first moment of the full goal-count distribution, whereas the latter matches the zero-event probability and therefore the binary event G≥1. Neither specification reproduces the entire Poisson–binomial law of *G*.

For binary event prediction, we evaluate probabilistic performance using the logarithmic score and the Brier score, both of which are standard proper scoring rules for probabilistic forecasts [16,17,18,19]:

(17) $$\mathrm{LogLoss}=-\frac{1}{m}\sum_{j=1}^{m}\left[y_{j}\log\left({\hat{p}}_{j}\right)+\left(1-y_{j}\right)\log\left(1-{\hat{p}}_{j}\right)\right],$$

(18) $$\mathrm{Brier}=\frac{1}{m}\sum_{j=1}^{m}{\left(y_{j}-{\hat{p}}_{j}\right)}^{2},$$

where $y_{j}\in \left\{0,1\right\}$ denotes the observed event indicator for observation *j*, ${\hat{p}}_{j}$ is the predicted probability of the event, and *m* is the number of team–match observations used for evaluation.

### 2.5. Calibration Metrics and Inference

Calibration is assessed using the Expected Calibration Error (ECE), the Integrated Calibration Index (ICI), and reliability diagrams [20,21]. The principal event in the main text is G≥1 because $xG^{+}$ is designed as a scoring-certainty measure. The complementary event G=0 is reported as a supplementary calibration analysis.

For the ECE, predicted probabilities are partitioned into B=10 approximately equal-frequency bins using rank-based quantile binning. When tied, predicted probabilities near bin boundaries may be split across adjacent bins to preserve approximately equal bin sizes. No additional tie-adjustment procedure was applied. Within each bin, we compute the mean predicted probability and the observed frequency, and the ECE is defined as the weighted average of the absolute differences between these quantities. This standard binning scheme does not introduce systematic bias, although, as with any discretization-based calibration measure, the resulting value depends on the choice of *B*. To assess robustness, we repeated the analysis with several alternative bin counts. The qualitative ordering of the models remained unchanged (Table A1). The ICI is computed from a smoothed calibration curve obtained by LOESS smoothing the binary outcome with respect to the predicted probability. We use a local quadratic fit (degree =2) with span =0.75 and no trimming. The ICI is defined as the mean absolute deviation between the LOESS-fitted curve and the identity line evaluated at the observed predicted probabilities.

To account for dependence between the two teams in the same match, inference is based on a nonparametric bootstrap that resamples matches (blocks) with replacement. Each bootstrap sample consists of 1446 matches, preserving the pairing of the two-team match observations within each match. For each resample, all metrics (log-loss, Brier, ECE, and ICI) are recomputed. Reported confidence intervals are based on the empirical 2.5% and 97.5% quantiles of 1000 bootstrap replicates.

## 3. Theoretical Results

This section distinguishes between exact structural identities, approximation-based results, and interpretive consequences. Exact statements are given in theorems and propositions, whereas approximation results are stated explicitly as second-order expansions or asymptotic representations. The section states the main theoretical properties of $xG^{+}$ and clarifies its relationship with total xG, shot concentration, and entropy. The results are intended primarily to provide a clear structural characterization of $xG^{+}$ in the present setting. While several arguments rely on standard tools such as convexity, majorization, and Taylor expansions, collecting them in this context helps clarify the relationships among scoring certainty, concentration, and Poisson-based summaries. Proofs are collected in Appendix D.

**Theorem 1**  (Dominance of $xG^{+}$ over xG)**.** *For any shot probability vector $p=(p_{1},\ldots ,p_{n})$ with $p_{i}\in \left[0,1\right)$,*

(19) $$xG^{+}\geq xG.$$

*Equality holds if and only if $p_{i}=0$ for all i=1,…,n.*

**Proof.**  The proof is provided in Appendix D.1.    □

**Theorem 2**  (Minimum of $xG^{+}$ for fixed mean)**.** *Fix n∈N and let $p=(p_{1},\ldots ,p_{n})$ satisfy $p_{i}\in \left[0,1\right)$ and*

$$\sum_{i=1}^{n}p_{i}=\mu ,\;0\leq \mu <n.$$

*The quantity*

$$xG^{+}\left(p\right)=\sum_{i=1}^{n}-\log\left(1-p_{i}\right)$$

*is minimized when all shot probabilities are equal, i.e.,*

$$p_{1}=\cdots =p_{n}=\frac{\mu }{n}.$$

*The minimum value is*

(20) $$xG_{\min}^{+}=-n\log\left(1-\frac{\mu }{n}\right).$$

**Proof.**  The proof is provided in Appendix D.2.    □

**Remark 1**  (Practical range)**.** *In football applications, the number of shots n may be large, and hence the total expected goals μ=xG can exceed 1 without restriction. The constraint μ<n is automatically satisfied because $p_{i}<1$ for all shots.*

**Proposition 1**  (Cumulative-hazard interpretation of $xG^{+}$)**.** *Let G denote the total number of goals generated by a shot profile. Then*

(21) $$P\left(G=0\right)=e^{-xG^{+}}.$$

*Equivalently,*

(22) $$P\left(G\geq 1\right)=1-e^{-xG^{+}}.$$

*Thus, $xG^{+}$ can be interpreted as the cumulative hazard associated with the event of scoring at least one goal.*

**Proof.**  The proof is provided in Appendix D.3.    □

**Theorem 3**  (Schur-convexity of $xG^{+}$)**.** *Let $p=(p_{1},\ldots ,p_{n})$ denote shot-level scoring probabilities with $p_{i}\in \left[0,1\right)$ and fixed sum*

$$\sum_{i=1}^{n}p_{i}=\mu .$$

*Define*

$$xG^{+}\left(p\right)=\sum_{i=1}^{n}-\log\left(1-p_{i}\right).$$

*Then $xG^{+}\left(p\right)$ is a Schur-convex function of p. In particular, if p majorizes q (denoted p≻q), then*

$$xG^{+}\left(p\right)\geq xG^{+}\left(q\right).$$

**Proof.**  The proof is provided in Appendix D.4.    □

**Theorem 4**  (Series representation and second-order approximation)**.** *For any shot profile p,*

(23) $$xG^{+}-xG=\sum_{i=1}^{n}\sum_{k=2}^{\infty }\frac{p_{i}^{k}}{k}.$$

*In particular, if the shot probabilities are sufficiently small, then*

(24) $$xG^{+}-xG=\frac{1}{2}\sum_{i=1}^{n}p_{i}^{2}+O\;\left(\sum_{i=1}^{n}p_{i}^{3}\right).$$

*Equivalently,*

(25) $$xG^{+}-xG\approx \frac{xG^{2}}{2N_{\mathrm{eff}}}.$$

**Proof.**  The proof is provided in Appendix D.5.    □

Unlike the dominance and Schur-convexity results, this representation yields an approximation whose accuracy depends on the magnitude of the shot probabilities.

**Remark 2**  (Rényi entropy interpretation)**.** *For shot profiles with xG>0, let $w_{i}=p_{i}/xG$ denote normalized shot probabilities so that $\sum_{i=1}^{n}w_{i}=1$. The effective number of shots can be written as*

$$N_{\mathrm{eff}}=\frac{1}{\sum_{i=1}^{n}w_{i}^{2}}.$$

*The quantity*

$$H_{2}=-\log\;\left(\sum_{i=1}^{n}w_{i}^{2}\right)$$

*is the Rényi entropy of order two, which implies*

$$N_{\mathrm{eff}}=e^{H_{2}}.$$

*Consequently, the second-order approximation derived in Theorem 4 can be written as *

$$xG^{+}-xG\approx \frac{xG^{2}}{2e^{H_{2}}}.$$

*Thus, the discrepancy between $xG^{+}$ and xG is inversely related to the effective number of scoring opportunities implied by the Rényi entropy. Profiles with low entropy (highly concentrated shot probabilities) produce larger curvature of the aggregated Bernoulli system, whereas diffuse profiles with high entropy yield values of $xG^{+}$ close to total xG. The appearance of Rényi entropy of order two is natural in this setting because the leading correction term in the expansion of $xG^{+}$ depends on $\sum_{i}p_{i}^{2}$, i.e., on the second moment of the normalized shot probabilities. Since $H_{2}=-\log\left(\sum_{i}w_{i}^{2}\right)$, the curvature term $xG^{+}-xG$ is directly governed by Rényi entropy rather than by Shannon entropy. This normalized entropy representation is defined only when xG>0. In the boundary case xG=0, all shot probabilities satisfy $p_{i}=0$, so $xG^{+}=0$, but the normalized weights and the associated entropy descriptors are treated as undefined.*

**Theorem 5**  (Log-loss optimality of $xG^{+}$)**.** *Let G denote the total number of goals generated by the shot profile. Consider the event Y=1(G≥1) and a shot probability vector $p=(p_{1},\ldots ,p_{n})$. Let*

(26) $$p^{*}=P\left(G\geq 1\right)=1-\prod_{i=1}^{n}\left(1-p_{i}\right).$$

*Among all predictors of the form*

(27) $$\hat{p}\left(\lambda \right)=1-e^{-\lambda },$$

*i.e., within the class of Poisson-induced predictors, the expected log-loss*

(28) $$L\left(\lambda \right)=E[l\left(\hat{p}\left(\lambda \right),Y\right)]$$

*is minimized at*

(29) $$\lambda =xG^{+}.$$

**Proof.**  The proof is provided in Appendix D.7.    □

This result shows that $xG^{+}$ is the log-loss optimal Poisson-equivalent rate for predicting the event of scoring at least one goal with the log-loss criterion. In contrast, xG represents the mean of the goal distribution and is therefore optimal for expected scoring volume rather than scoring certainty.

**Figure 3:**
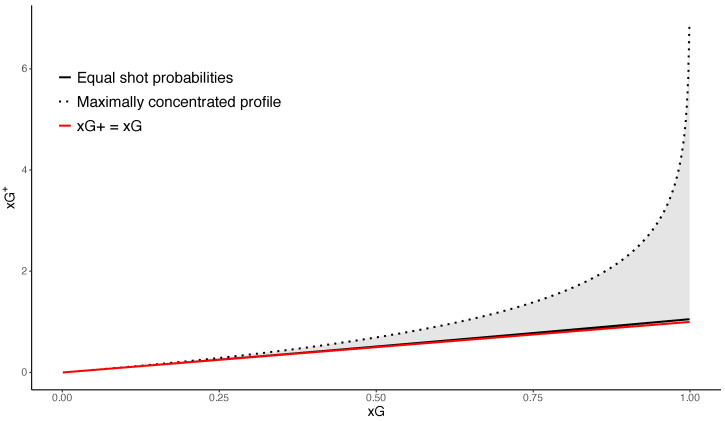
Feasible region of $\left(xG,xG^{+}\right)$ for a fixed number of shots n=10. The solid curve shows the minimal value of $xG^{+}$ obtained when shot probabilities are equal ($p_{i}=xG/n$). The dotted curve corresponds to the most concentrated admissible profile for a given xG and fixed *n*. For xG<1, this reduces to allocating all mass to a single shot. For xG≥1, the upper boundary is approached by profiles with one or more shot probabilities arbitrarily close to one. The shaded region, therefore, represents all possible values of $xG^{+}$ compatible with a given total xG. The red line shows the theoretical lower bound $xG^{+}=xG$ established in Theorem 1. The upper boundary corresponds to the limiting case of maximal concentration and is finite only for xG<1.

Taken together, these results show that $xG^{+}$ possesses several structural properties absent from standard expected goals. It dominates xG, depends monotonically on shot concentration, admits a normalized Rényi-2 interpretation, and arises as the log-loss optimal Poisson rate for predicting the event of scoring at least one goal. Thus, $xG^{+}$ naturally quantifies certainty about scoring at least once, while xG captures expected scoring volume. These theoretical results also imply geometric constraints for the relationship between xG and $xG^{+}$. Figure 3 illustrates the feasible region for $\left(xG,xG^{+}\right)$ when the number of shots is fixed. The lower boundary corresponds to the equal-probability profile, which minimizes $xG^{+}$ for fixed total xG, while the upper boundary corresponds to maximally concentrated shot profiles. The upper bound $xG^{+}=-\log\left(1-xG\right)$ is valid only for xG<1. For xG≥1, the quantity $xG^{+}$ becomes unbounded as one of the shot probabilities approaches one.

## 4. Empirical Results

The empirical analyses in this section illustrate how the theoretical properties of $xG^{+}$ appear in the analyzed football dataset. The goal is not to provide a universal predictive benchmark but to quantify the practical differences between exact Bernoulli aggregation and mean-matched Poisson approximation.

### 4.1. Data

The empirical analysis is based on publicly available football event data from StatsBomb Open Data, which provides shot-level expected goals (xG) values and shot outcomes. We restrict the analysis to regular time (90 min plus stoppage time); extra time is not present in league matches and is therefore not considered. Penalty kicks are included as standard shots with their associated xG values. Shot-level probabilities $p_{i}$ are taken directly from the xG values provided in the dataset, and no additional recalibration of the xG model is performed. This allows us to isolate the effect of aggregation rather than model specification. Accordingly, the empirical interpretation of $xG^{+}$ is conditional on the adequacy of these externally supplied shot-level probabilities. We do not independently validate or recalibrate the underlying xG model for the competitions and seasons analyzed here. Therefore, the empirical results should be interpreted as conditional on the quality of the underlying xG inputs rather than as a validation of the xG model itself. This also means that $xG^{+}$ does not provide robustness to misspecification in the shot-level probabilities; it propagates the quality of the underlying xG inputs through a different aggregation rule. Each shot is treated as a Bernoulli trial with success probability $p_{i}$, and all quantities are aggregated at the team–match level. Shots with xG=0 are retained for completeness. In the empirical dataset, no team–match observation with total xG=0 occurred. Operationally, if such a case were present, the quantities based on normalized weights, including $N_{\mathrm{eff}}$ and entropy measures, would be treated as undefined and omitted from summaries requiring these descriptors. All quantities (xG, $xG^{+}$, $N_{\mathrm{eff}}$, etc.) are computed at the team–match level. No matches with missing or inconsistent event data were observed in the dataset. No additional filtering or smoothing of shot probabilities was applied.

**Figure 4:**
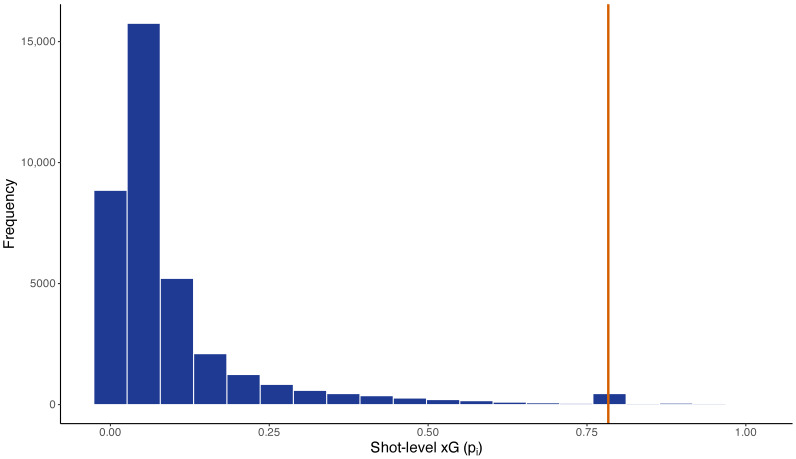
Empirical distribution of shot-level expected goal probabilities ($p_{i}=xG_{i}$). A visible spike at $p_{i}\approx 0.78$ corresponds to penalty kicks.

The empirical analysis is based on publicly available football event data from four major European men’s leagues in the 2015/2016 season: La Liga, the Premier League, Serie A, and the Bundesliga. The resulting sample comprises 1446 matches, yielding 2892 team–match observations and 36,905 shots. To assess the validity of the small-probability approximation used in Theorem 4, we examine the empirical distribution of shot-level probabilities $p_{i}$ (Figure 4). The distribution is strongly right-skewed, with most shots having low scoring probabilities. This supports the accuracy of the second-order approximation, as higher-order terms remain small for most observations.

The theoretical results developed in this paper are model-free and apply to any set of shot-level scoring probabilities, whereas the empirical findings remain conditional on the competitions, seasons, and xG inputs analyzed here.

### 4.2. Descriptive Comparison of xG and $xG^{+}$

**Figure 5:**
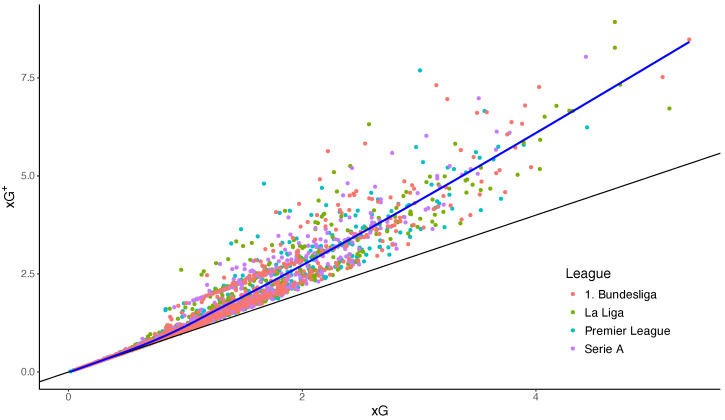
Empirical relationship between total expected goals (xG) and the Poisson-equivalent scoring rate ($xG^{+}$). The diagonal line denotes $xG^{+}=xG$. All observations lie above this bound, consistent with Theorem 1. Deviations from the diagonal reflect the concentration of shot probabilities: profiles with a few high-probability chances yield larger values of $xG^{+}$ relative to total xG.

The first empirical objective is to compare total xG and $xG^{+}$ across team-matches. By Theorem 1, every observation must satisfy $xG^{+}\geq xG$. Empirically, this inequality is often strict. Games with highly concentrated chance creation tend to lie substantially above the identity line, whereas diffuse profiles with many small-probability shots remain much closer to it. This pattern indicates that total xG may understate certainty about scoring at least once when shot-level risk is concentrated. Table 2 reports descriptive statistics for the analyzed dataset. On average, realized goals closely match total xG, while $xG^{+}$ systematically exceeds xG, reflecting the effect of shot concentration on scoring certainty. To illustrate the empirical relationship between xG and $xG^{+}$, Figure 5 plots match-level observations of both quantities. The Poisson-equivalent rate $xG^{+}$ is systematically larger than the total xG. The discrepancy tends to increase with offensive potential (higher xG), reflecting the effect of shot concentration. When scoring probability is concentrated in a small number of high-quality chances, standard xG underestimates the probability of scoring at least one goal.

### 4.3. Approximation by the Effective Number of Shots

Theorem 4 implies the approximation

$$xG^{+}-xG\approx \frac{xG^{2}}{2N_{\mathrm{eff}}}.$$

**Figure 6:**
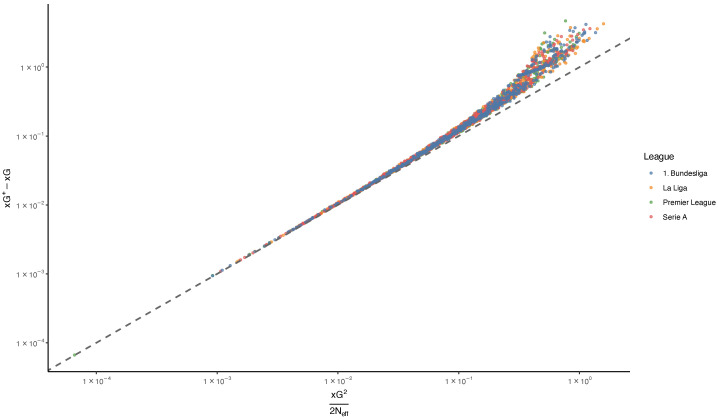
Observed values of $xG^{+}-xG$ versus the second-order approximation $xG^{2}/\left(2N_{\mathrm{eff}}\right)$. Each point represents one team–match observation, and the dashed line denotes the identity line. Most observations lie close to the identity line, indicating good agreement between the observed gap and its second-order approximation.

This formula is important because it connects a nonlinear certainty measure to a simple concentration statistic. Figure 6 compares the observed values of $xG^{+}-xG$ with this second-order approximation. The majority of observations lie close to the identity line, indicating that the approximation provides a good description of the discrepancy between xG and $xG^{+}$ across a wide range of match situations. Deviations from the identity line reflect higher-order terms neglected in the second-order expansion and become more pronounced for matches with larger shot probabilities or stronger concentration. In our dataset, over 92% of shot probabilities are below 0.3, and about 3% exceed 0.5, which explains the generally good performance of the second-order approximation.

### 4.4. Calibration of Scoring Probabilities

The most direct event-level interpretation of $xG^{+}$ concerns the probability of scoring at least one goal. Using a Poisson model with rate xG, the predicted scoring probability is $1-e^{-xG}$. With $xG^{+}$, the corresponding probability is $1-e^{-xG^{+}}$, which exactly matches the scoreless-match probability implied by the shot-level Bernoulli process. The central empirical question is not whether a new predictor can outperform an unrelated benchmark but how large the practical discrepancy is between the exact Bernoulli aggregation implied by the shot-level probabilities and the simpler Poisson approximation based only on total xG. Because $xG^{+}$ matches the scoreless-match probability of the underlying shot-level Bernoulli system by construction, it is theoretically aligned with the event G≥1 at the model level. However, the empirical magnitude of any calibration improvement remains data-dependent and may be affected by sampling variability, model misspecification in the shot-level probabilities, and residual dependence between shots. (Since G≥1 and G=0 are complementary events, the log-loss and Brier scores are identical for both targets. For brevity, only the results for G≥1 are reported. In contrast, calibration metrics such as the ECE or ICI depend on the distribution of predicted probabilities and may therefore differ when modeling G≥1 versus G=0.)

Table 3 reports scoring-rule and calibration metrics for predicting the event of scoring at least one goal. Across all reported point estimates, the specification based on $xG^{+}$ performs more favorably than the Poisson model based on total xG. In particular, the log-loss decreases from 0.5040 to 0.4850 and the Brier score from 0.1670 to 0.1610. Calibration metrics show an even stronger pattern: both the expected calibration error (ECE) and the integrated calibration index (ICI) are substantially smaller for $xG^{+}$. In relative terms, the $xG^{+}$ specification improves log-loss by 3.77% and the Brier score by 3.59%, while the ECE and ICI are reduced by 63.62% and 75.67%, respectively. Bootstrap analysis of the metric differences indicates positive average improvements for all measures. Moreover, the bootstrap 95% confidence intervals for the log-loss, Brier score, ECE, and ICI lie above zero, indicating that the observed advantage of $xG^{+}$ over the mean-matched Poisson approximation is stable across the analyzed dataset. These results are consistent with the theoretical construction: because $xG^{+}$ is derived from the exact scoreless probability in the shot-level Bernoulli system, it is naturally better aligned with the binary event G≥1 than the simpler Poisson approximation based only on total xG. This comparison is best interpreted as quantifying the practical difference between exact Bernoulli aggregation and a Poisson approximation based only on the mean.

**Figure 7:**
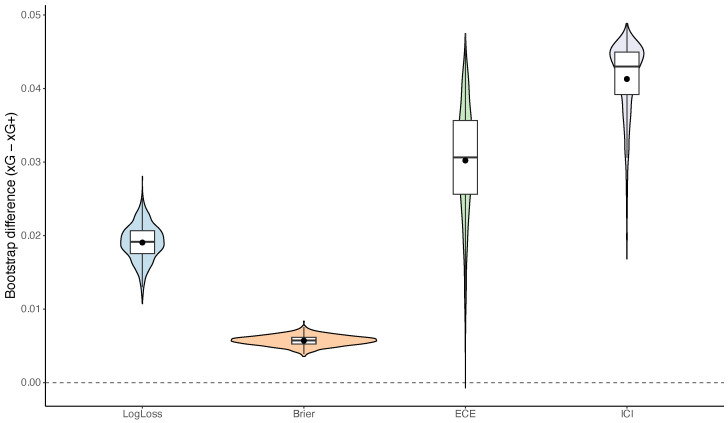
Bootstrap distributions of the differences in scoring-rule and calibration metrics between the Poisson models based on xG and $xG^{+}$.

Figure 7 shows the bootstrap distributions of the metric differences between the two specifications. The plotted quantity is $\Delta ={\mathrm{metric}}_{xG}-{\mathrm{metric}}_{xG^{+}}$, so positive values indicate more favorable performance of the $xG^{+}$ model. All four distributions are concentrated above zero, which is consistent with the positive bootstrap mean differences and confidence intervals reported in Table 3. The gains in the log-loss and Brier score are relatively modest but stable, whereas the improvements in the ECE and ICI are larger in magnitude. Overall, these bootstrap results reinforce the descriptive pattern reported in Table 3: in the analyzed dataset, the $xG^{+}$-based probability is systematically better aligned with the binary event G≥1 than the mean-matched Poisson approximation based only on total xG. This pattern is consistent with the theoretical construction, which implies that $xG^{+}$ is more directly aligned with scoring certainty for the event G≥1 than a Poisson model parameterized solely by total xG.

**Figure 8:**
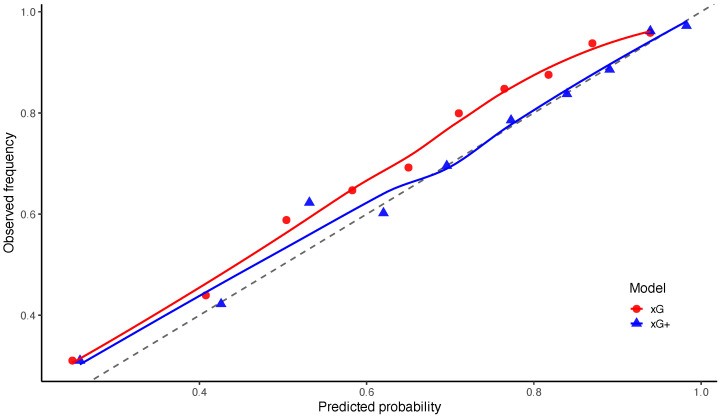
Calibration of predicted probabilities for the event of scoring at least one goal (G≥1).

**Figure A1:**
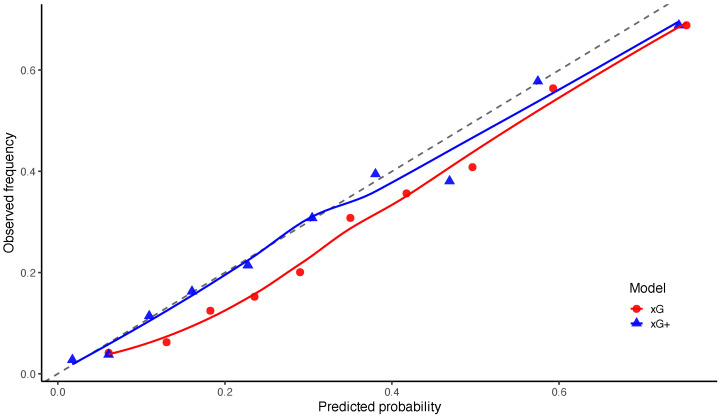
Calibration curves for the predicted probability of the scoreless outcome (G=0).

Figure 8 presents the reliability diagram for the event G≥1. The complementary reliability plot for G=0 is placed in Figure A1. Both models appear reasonably well calibrated overall. However, the $xG^{+}$ specification tends to lie closer to the diagonal of perfect calibration, particularly in the intermediate probability range, consistent with the improvements observed in the scoring-rule and calibration metrics.

### 4.5. Poisson Approximation Errors

A separate but related question is how much error is introduced by using Pois(xG) instead of exact Poisson–binomial probabilities. For the scoreless-match event, define

$${\mathrm{err}}_{P0}=e^{-xG}-P\left(G=0\right),$$

and for the event G≥2, define

$${\mathrm{err}}_{P2+}=\left[1-e^{-xG}\left(1+xG\right)\right]-P\left(G\geq 2\right).$$

**Figure 9:**
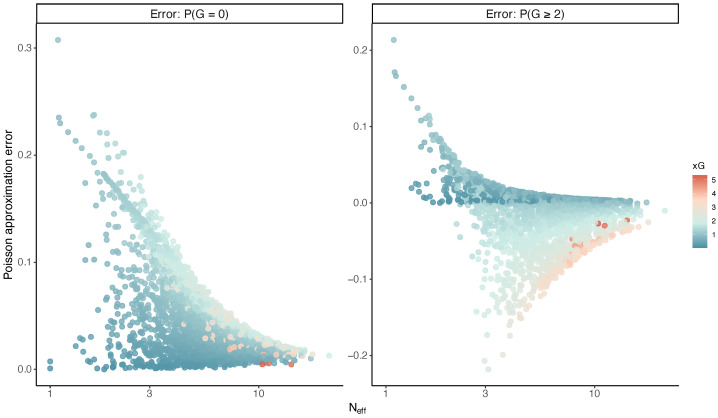
Poisson approximation error as a function of shot concentration. The left panel shows the error for the probability of no goal, P(G=0), and the right panel for the probability of scoring at least two goals, P(G≥2).

These quantities isolate the error caused by collapsing the full shot profile into a single Poisson rate. Figure 9 shows that the Poisson approximation error decreases with increasing $N_{\mathrm{eff}}$. This confirms that the Poisson model performs well for diffuse shot profiles but deteriorates when shot probabilities are highly concentrated. This pattern is consistent with Theorem 4, which shows that approximation error is driven by the second moment of shot probabilities. This comparison is descriptive and pertains to the analyzed dataset, whereas the theoretical mechanism is general.

## 5. Discussion

This paper shows that the informational structure of shot-level scoring probabilities matters in football and that total xG alone does not provide a complete probabilistic description of offensive performance. In particular, xG is an appropriate summary of expected scoring volume, but it does not encode how scoring chances are distributed across shots. Once that structural information is taken into account, the probability of scoring at least one goal can differ substantially even when total xG is unchanged.

A useful way to interpret the proposed framework is as a comparison between alternative Poisson-induced summaries of the same shot-level Bernoulli process. The classical quantity xG induces a Poisson approximation through mean matching and is therefore appropriate when interest centers on expected goal volume. The proposed $xG^{+}$ induces a different Poisson representation through matching the scoreless probability and is therefore appropriate when interest centers on the event of scoring at least once. In this sense, the framework does not reject Poisson-based approximation; rather, it shows that different Poisson-induced reductions are appropriate for different predictive targets.

The proposed quantity $xG^{+}$ addresses this limitation by encoding certainty about scoring at least once through the exact probability of a scoreless match. It should not be viewed as a new event probability but as an additive reparameterization of the exact event probability that is analytically convenient for studying concentration and Poisson-induced approximations. The quantity is mathematically simple, additive across shots, and directly linked to a Poisson-equivalent representation. It therefore preserves the interpretability that has made xG attractive while incorporating risk structure that the mean alone cannot capture.

The theoretical value of the paper lies less in technical novelty at the level of proof machinery than in the coherent organization and interpretation of the structural properties of $xG^{+}$ in the present setting. The theoretical results clarify why this matters. First, $xG^{+}$ always exceeds or equals xG. Thus, xG can be viewed as a lower bound on the Poisson-equivalent rate associated with scoring at least once. Second, the second-order expansion shows that the gap between $xG^{+}$ and xG is driven by the sum of squared shot probabilities and therefore by concentration. Third, Jensen’s inequality establishes that for fixed total xG and fixed shot count, $xG^{+}$ is minimized by the equal-probability profile. Because these results rely on standard tools such as convexity, majorization, and Taylor expansion, their role is primarily structural and interpretive: they clarify how scoring certainty depends on the underlying Bernoulli profile. Together, these results explain why concentrated shot profiles systematically generate larger $xG^{+}$/xG discrepancies.

The empirical findings are broadly consistent with these theoretical arguments. Concentrated profiles tend to produce larger values of $xG^{+}-xG$, and conventional Pois(xG) approximations can be miscalibrated for the event of scoring at least one goal. However, this empirical comparison should not be interpreted as the validation of a genuinely new predictive method. Because $xG^{+}$ is constructed from the exact scoreless probability within the shot-level Bernoulli system, its superiority over the mean-matched Poisson approximation for the binary event G≥1 is largely built into the construction. The empirical contribution, therefore, lies mainly in illustrating the practical magnitude of the discrepancy between exact Bernoulli aggregation and a Poisson approximation based only on the mean. This is not a trivial modeling detail: in applications such as tactical interpretation, match simulation, or probability communication, the distinction between expected scoring volume and scoring certainty can be substantively important.

The paper also highlights the usefulness of concentration descriptors. The effective number of shots provides a direct link between the theory and the data by explicitly entering the second-order approximation. The exact analytical link is with the second moment and its normalized reformulation via the Rényi entropy of order 2, whereas Shannon entropy plays only a secondary descriptive role. In this sense, football provides an empirically accessible setting for a broader class of aggregated Bernoulli systems in which first moments fail to capture meaningful information about concentration.

As an additional exploratory benchmark, we compared Pois$\left(xG^{+}\right)$ with simple logistic models for the event G≥1. In the pooled analysis, Pois$\left(xG^{+}\right)$ achieved the lowest log-loss and Brier score, while the best logistic alternatives remained close (Appendix E, Table A2). The same qualitative pattern was observed in the league-specific robustness check (Appendix E, Table A3). This suggests that the practical usefulness of $xG^{+}$ is not driven solely by comparisons with Pois(xG) and that part of the same event-level information can be captured by simple, data-driven binary predictors.

Several limitations should be acknowledged. A central limitation of the present framework is that it relies on a conditional Bernoulli working model at the shot level. This entails two non-trivial assumptions: first, that shot-level xG values can be interpreted as probability-like Bernoulli parameters, and second, that shot outcomes are conditionally independent given those values. Neither assumption is innocuous. In practice, xG values are estimated model outputs rather than true probabilities, and football shots may remain temporally and tactically dependent because of possession sequences, score effects, match context, or latent team-level factors. Consequently, the theoretical results in this paper should be read as characterizing the behavior of aggregated Bernoulli risk with a transparent benchmark model rather than with a complete generative description of match dynamics.

A related limitation is that $xG^{+}$ depends entirely on the quality of the underlying shot-level probabilities. In the empirical analysis, these probabilities are taken directly from the StatsBomb xG model without additional recalibration. Therefore, any miscalibration, omitted-variable bias, or structural misspecification in the underlying xG inputs will generally be inherited by $xG^{+}$ rather than corrected by it. Moreover, because the mapping p↦−log(1−p) is nonlinear and becomes increasingly steep as *p* approaches one, misspecification in high-probability shots may have a disproportionately large effect on $xG^{+}$. In this sense, $xG^{+}$ should be interpreted as conditionally valid given the adequacy of the underlying shot-level probability model, rather than as a quantity that corrects for deficiencies in those inputs.

A further limitation concerns empirical scope. Although using publicly available StatsBomb event data from multiple major European leagues provides a broader basis for illustration than a single-team analysis, the empirical findings should still not be treated as universal validation across all competitions, seasons, or xG models. In particular, calibration metrics and smooth reliability curves remain sensitive to the composition of the analyzed sample and to the underlying quality of the xG inputs. The empirical results should therefore be interpreted as a broad illustration of the framework within the selected dataset rather than as a definitive benchmark for football forecasting in general.

Several directions for future work follow naturally. These include extending the empirical analysis to additional leagues, seasons, and xG models; examining the effect of shot-outcome dependence; studying robustness to misspecification in shot-level probabilities; and comparing the proposed framework with broader classes of binary-event prediction models. More broadly, the same distinction between expected event count and certainty of at least one event arises in other aggregated Bernoulli settings, including reliability analysis, medical risk modeling, and related risk-aggregation problems.

## 6. Conclusions

Total expected goals summarize offensive production through a shot-level Bernoulli system. This is useful but incomplete. The quantity $xG^{+}$ complements xG by encoding certainty about scoring at least once through the exact scoreless-match probability. It is additive across shots, always greater than or equal to xG, and structurally linked to concentration through the second moment of shot probabilities. The effective number of shots and Rényi entropy of order two provide convenient normalized reformulations of this concentration structure, whereas Shannon entropy plays only a secondary descriptive role.

The central contribution of the paper is therefore not the mere observation that $xG^{+}$ is a monotone transformation of the exact probability of scoring at least once. Rather, it is the identification and interpretation of $xG^{+}$ as an additive, event-specific summary of aggregated Bernoulli risk, together with a structural characterization of how heterogeneity in shot probabilities affects scoring certainty. In this sense, the contribution is interpretive and structural rather than primarily technical.

From the perspective of Poisson-induced approximations, xG and $xG^{+}$ summarize different aspects of the same underlying heterogeneous Bernoulli system. The standard Pois(xG) representation is obtained by matching the expected number of goals, whereas Pois$\left(xG^{+}\right)$ is obtained by matching the probability of the zero-goal event. Accordingly, the associated Poisson parameter should be interpreted differently in the two cases: as a mean-goal parameter for xG, and as an event-specific zero-event-matching rate for $xG^{+}$.

The main substantive implication is that offensive performance should not be reduced to expected goal volume alone. Two matches with identical xG may nevertheless imply different probabilities of scoring at least once because their shot-level probabilities are distributed differently. In this sense, xG and $xG^{+}$ should be viewed as complementary summaries: the former measures expected scoring volume, whereas the latter targets scoring certainty. In practical terms, xG answers the question “How many goals are expected?”, while $xG^{+}$ answers “How likely is it that at least one goal will be scored?” Large values of $xG^{+}$ should therefore be interpreted as indicating near-certainty of at least one goal, not as implying a large expected goal count.

The empirical analysis is consistent with this interpretation, but it should be understood primarily as an illustration of the practical consequences of replacing a mean-matched Poisson approximation with exact Bernoulli aggregation for the event G≥1. In the analyzed dataset, the $xG^{+}$-based probability yielded more favorable scoring-rule and calibration results than the standard Pois(xG) specification, and an additional exploratory benchmark against simple logistic alternatives led to the same qualitative conclusion. These findings strengthen the practical interpretation of the framework, but they do not constitute a separate claim of universal predictive superiority.

More broadly, the proposed framework is not specific to football. Similar aggregated Bernoulli structures arise in reliability analysis, medicine, and other risk-aggregation settings, where the mean alone may not adequately describe the probability of at least one event. From this perspective, $xG^{+}$ provides a football-based illustration of a more general principle concerning event-level certainty under heterogeneous risk conditions.

Several directions for future work follow naturally. These include extending the empirical analysis to additional leagues, seasons, and xG models; examining the effect of shot-outcome dependence; studying robustness to misspecification in shot-level probabilities; and comparing the proposed framework with broader classes of binary-event prediction models. Taken together, these results suggest that when interest centers on the occurrence of at least one event, structurally informed summaries of aggregated risk may be more informative than the mean alone.

## Figures and Tables

**Table 1:** Summary of the motivating example with two shot profiles of identical total xG=1.5.

Profile	xG	${\mathrm{xG}}^{+}$	P(G=0)	P(G≥1)	$N_{\mathrm{eff}}$
Concentrated	1.5	2.477	0.084	0.916	2.530
Diffuse	1.5	1.580	0.206	0.794	15.000

**Table 2:** Descriptive statistics of the main match-level quantities.

Variable	Mean	Median	SD	Min	Max
Goals	1.311	1.000	1.224	0.000	10.000
xG	1.281	1.142	0.773	0.015	5.313
$xG^{+}$	1.656	1.335	1.229	0.016	8.927
$xG^{+}-xG$	0.375	0.149	0.535	0.000	4.684
P(G≥1) (ScoreProb)	0.695	0.737	0.227	0.015	1.000
P(G=0)	0.305	0.263	0.227	0.000	0.985
$N_{\mathrm{eff}}$	6.055	5.638	2.757	1.000	21.708
Entropy (H)	1.990	2.031	0.453	0.000	3.262

**Table 3:** Scoring-rule and calibration metrics for predicting the event G≥1. Bold values indicate the better-performing model for each metric; since all metrics are minimized, lower values are better.

Model	Logloss ↓	Brier ↓	ECE ↓	ICI ↓
xG	0.5040	0.1670	0.0602	0.0596
$xG^{+}$	**0.4850**	**0.1610**	**0.0219**	**0.0145**
Improvement (in %)	3.77	3.59	63.62	75.67
Boot. mean diff.	0.019	0.006	0.030	0.041
Boot. SD	0.002	0.001	0.008	0.005
Bootstrap 95% CI	(0.014,0.024)	(0.004,0.007)	(0.013,0.043)	(0.028,0.047)

## Data Availability

The event data analyzed in this study are publicly available through StatsBomb Open Data.

## Abbreviations

The following abbreviations are used in this manuscript:

xG	Expected goals
xG^+^	Poisson-equivalent scoring rate for G≥1
ECE	Expected calibration error
ICI	Integrated calibration index

## Appendix A. Supplementary Calibration

Figure A1 presents calibration curves for predicting the scoreless outcome (G=0). The curves compare the Poisson model parameterized by total xG with the specification based on $xG^{+}$. Points represent bin-level observed frequencies, while the diagonal line corresponds to perfect calibration. Deviations from the diagonal indicate systematic miscalibration of predicted probabilities. This figure is particularly relevant because $xG^{+}$ is defined through the scoreless probability, $xG^{+}=-\log P\left(G=0\right)$, so the calibration of predictions for the event G=0 directly reflects the structural properties of the proposed measure.

## Appendix B. Sensitivity of the ECE to the Number of Bins

The expected calibration error depends on the number of calibration bins used. To verify the robustness of the results, we recomputed the ECE using several alternative bin counts. Table A1 reports the resulting values for both models. The calibration advantage of $xG^{+}$ over xG is robust across bin counts. Although the absolute value of the ECE increases with the number of bins, the relative ordering of the models remains unchanged.

**Table A1:** Sensitivity of the ECE to the number of bins used in the calibration calculation.

Bins (*B*)	xG	${\mathrm{xG}}^{+}$
5	0.060	0.015
10	0.060	0.022
15	0.060	0.029
20	0.061	0.028

## Appendix C. Additional Remarks and Propositions

**Remark A1** (High-probability shots)**.** *The function ϕ(p)=−log(1−p) diverges as p→1. Consequently, $xG^{+}$ becomes large when shot probabilities are close to one, even if the total xG remains moderate. This behavior should not be interpreted as implying a very large expected number of goals. Rather, it reflects the fact that the probability of a scoreless outcome becomes very small, so scoring at least once is nearly certain. Although such extreme shot probabilities are rare in practice, this property helps clarify that $xG^{+}$ is a certainty-oriented transformation, not a volume-based scoring measure.*

**Remark A2** (Additivity)**.** *The quantity $xG^{+}$ is additive across independent collections of shots. Suppose that the shot profile p can be partitioned into two disjoint subsets A and B that are probabilistically independent. Then*

$$xG^{+}\left(A\cup B\right)=xG^{+}\left(A\right)+xG^{+}\left(B\right),$$

*because the scoreless probabilities multiply:*

P(G=0∣A∪B)=P(G=0∣A)·P(G=0∣B).

*Taking negative logarithms yields the additive decomposition above. This property makes $xG^{+}$ naturally decomposable across halves, phases of play, or possession sequences.*

**Remark A3** (Second-order approximation and remainder)**.** *Using the expansion*

$$-\log\left(1-p\right)=p+\frac{p^{2}}{2}+\frac{p^{3}}{3}+\ldots ,$$

*we obtain*

$$xG^{+}-xG=\frac{1}{2}\sum_{i}p_{i}^{2}+R_{3},\;R_{3}=\sum_{i}\sum_{k\geq 3}\frac{p_{i}^{k}}{k}.$$

*If $\max_{i}p_{i}\leq \delta <1$, then*

$$0\leq R_{3}\leq \frac{1}{3}\sum_{i}\frac{p_{i}^{3}}{1-\delta }.$$

*Hence, the approximation is accurate when shot probabilities are not too large.*

**Proposition A1** (Variance representation of the $xG^{+}$ gap)**.** *Let G denote the total number of goals generated by the shot profile $p=(p_{1},\ldots ,p_{n})$. The Poisson–binomial distribution implies*

$$E\left[G\right]=xG,\;V\mathrm{ar}\left(G\right)=\sum_{i=1}^{n}p_{i}\left(1-p_{i}\right).$$

*Then the difference between $xG^{+}$ and xG satisfies*

(A1) $$xG^{+}-xG=\frac{1}{2}\left(xG-V\mathrm{ar}(G)\right)+R,$$

*where the remainder term satisfies*

$$R=O\;\left(\sum_{i=1}^{n}p_{i}^{3}\right).$$

**Proof.** The proof is provided in Appendix D.6.    □

**Remark A4.** 
*This result provides a variance-based interpretation of the $xG^{+}$ gap. When shot probabilities are highly concentrated, the variance of the Poisson–binomial goal distribution decreases relative to its mean, which increases the difference $xG^{+}-xG$. Conversely, diffuse shot profiles produce variance closer to the Poisson benchmark Var(G)=xG, and the gap between $xG^{+}$ and xG becomes small. This variance-based interpretation is approximate rather than exact because it relies on the second-order expansion of $xG^{+}-xG$.*

**Remark A5** (Shannon entropy as a secondary descriptor)**.** *For profiles with xG>0, Shannon entropy provides an additional descriptive measure of dispersion, but the exact analytical characterization of $xG^{+}-xG$ is governed by the second moment $\sum_{i}p_{i}^{2}$, equivalently by $N_{\mathrm{eff}}$ and Rényi entropy of order two. As above, this normalized entropy representation is undefined in the boundary case xG=0.*

**Proposition A2** (Kullback–Leibler interpretation of $xG^{+}$)**.** *Let G denote the total number of goals generated by the shot profile $p=(p_{1},\ldots ,p_{n})$ and let*

$$p^{*}=P\left(G\geq 1\right)=1-\prod_{i=1}^{n}\left(1-p_{i}\right)$$

*be the true probability of scoring at least one goal.*
*Consider a Poisson model with rate λ, which implies*

$$\hat{p}\left(\lambda \right)=1-e^{-\lambda }.$$

*Then the expected log-loss can be written as*

$$L\left(\lambda \right)=H\left(p^{*}\right)+D_{\mathrm{KL}}\;\left(\mathrm{Bern}\left(p^{*}\right)\;∥\;\mathrm{Bern}\left(\hat{p}\left(\lambda \right)\right)\right),$$

*where $H(p^{*})$ is the Shannon entropy of the Bernoulli variable and $D_{\mathrm{KL}}$ denotes the Kullback–Leibler divergence.*

*In particular, the divergence term is minimized at*

$$\lambda =xG^{+}.$$

**Proof.** The proof is provided in Appendix D.8.    □

**Remark A6.** 
*$xG^{+}$ is the Poisson-induced rate that minimizes the Kullback–Leibler divergence at the level of the binary event G≥1. Thus, it is the information-optimal Poisson-equivalent rate for this event, not for the full goal-count distribution. Consequently, $xG^{+}$ can be interpreted as the information-optimal Poisson-equivalent rate for the binary event of scoring at least one goal.*

## Appendix D. Proofs of Theoretical Results

### Appendix D.1. Proof of Theorem 1

By Equation (10),

$$xG^{+}=\sum_{i=1}^{n}\left(-\log(1-p_{i})\right).$$

Define

f(p)=−log(1−p)−p,p∈[0,1).

We have f(0)=0 and

$$f^{\prime }\left(p\right)=\frac{1}{1-p}-1=\frac{p}{1-p}\geq 0.$$

Hence *f* is nondecreasing on [0,1), which implies f(p)≥0 for all p∈[0,1). Therefore,

$$-\log\left(1-p_{i}\right)\geq p_{i}\;\mathrm{for}\;\mathrm{all}\;i.$$

Summing over *i* gives

$$xG^{+}=\sum_{i=1}^{n}-\log\left(1-p_{i}\right)\geq \sum_{i=1}^{n}p_{i}=xG.$$

Equality holds if and only if $f(p_{i})=0$ for every *i*, which occurs only at $p_{i}=0$. This proves the theorem.

### Appendix D.2. Proof of Theorem 2

Consider the function

ϕ(p)=−log(1−p),p∈[0,1).

Since

$$\phi^{\prime \prime }\left(p\right)=\frac{1}{{\left(1-p\right)}^{2}}>0,$$

*ϕ* is strictly convex. By Jensen’s inequality,

$$\frac{1}{n}\sum_{i=1}^{n}\phi \left(p_{i}\right)\geq \phi \;\left(\frac{1}{n}\sum_{i=1}^{n}p_{i}\right).$$

Because $\sum_{i}p_{i}=\mu$,

$$\frac{1}{n}\sum_{i=1}^{n}-\log\left(1-p_{i}\right)\geq -\log\left(1-\frac{\mu }{n}\right).$$

Multiplying both sides by *n* gives Equation (20). Equality holds if and only if all $p_{i}$ are equal.

### Appendix D.3. Proof of Proposition 1

By definition,

$$xG^{+}=-\log P\left(G=0\right).$$

Exponentiating both sides yields

$$P\left(G=0\right)=e^{-xG^{+}}.$$

Subtracting from one gives

$$P\left(G\geq 1\right)=1-e^{-xG^{+}}.$$

Hence, $xG^{+}$ has the standard cumulative-hazard representation for the event of scoring at least one goal.

### Appendix D.4. Proof of Theorem 3

Let ϕ(p)=−log(1−p) for p∈[0,1). Since

$$\phi^{\prime \prime }\left(p\right)=\frac{1}{{\left(1-p\right)}^{2}}>0,$$

the function ϕ is strictly convex. A classical result in majorization theory states that sums of convex functions,

$$F\left(p\right)=\sum_{i=1}^{n}\phi \left(p_{i}\right),$$

are Schur-convex [22]. Therefore, if p majorizes q, then

$$\sum_{i=1}^{n}\phi \left(p_{i}\right)\geq \sum_{i=1}^{n}\phi \left(q_{i}\right).$$

Substituting ϕ(p)=−log(1−p) gives

$$xG^{+}\left(p\right)\geq xG^{+}\left(q\right),$$

which proves the result.

### Appendix D.5. Proof of Theorem 4

Using the Taylor expansion

$$-\log\left(1-p\right)=p+\frac{p^{2}}{2}+\frac{p^{3}}{3}+\ldots ,\;\left|p\right|<1,$$

we obtain

$$-\log\left(1-p_{i}\right)-p_{i}=\sum_{k=2}^{\infty }\frac{p_{i}^{k}}{k}.$$

Summing over *i* yields Equation (23). Retaining the leading term gives Equation (24). Finally, Equation (13) implies

$$\sum_{i=1}^{n}p_{i}^{2}=\frac{xG^{2}}{N_{\mathrm{eff}}},$$

and substitution yields Equation (25).

### Appendix D.6. Proof of Proposition A1

From Theorem 4,

$$xG^{+}-xG=\sum_{i=1}^{n}\sum_{k=2}^{\infty }\frac{p_{i}^{k}}{k}.$$

Retaining the leading term gives

$$xG^{+}-xG=\frac{1}{2}\sum_{i=1}^{n}p_{i}^{2}+O\;\left(\sum_{i=1}^{n}p_{i}^{3}\right).$$

For the Poisson–binomial distribution,

$$V\mathrm{ar}\left(G\right)=\sum_{i=1}^{n}p_{i}\left(1-p_{i}\right)=\sum_{i=1}^{n}p_{i}-\sum_{i=1}^{n}p_{i}^{2}=xG-\sum_{i=1}^{n}p_{i}^{2}.$$

Hence

$$\sum_{i=1}^{n}p_{i}^{2}=xG-V\mathrm{ar}\left(G\right).$$

Substituting into the second-order approximation yields

$$xG^{+}-xG=\frac{1}{2}\left(xG-V\mathrm{ar}(G)\right)+O\;\left(\sum_{i=1}^{n}p_{i}^{3}\right).$$

### Appendix D.7. Proof of Theorem 5

Let Y=1(G≥1) denote the indicator of the event that at least one goal is scored. Define the true probability

$$p^{*}=P\left(Y=1\right)=1-P\left(G=0\right).$$

For a Poisson-type predictor parameterized by λ, the predicted probability of the event is

$$\hat{p}\left(\lambda \right)=1-e^{-\lambda }.$$

The log-loss for a single observation is

$$l\left(\hat{p}\left(\lambda \right),Y\right)=-\left[Y\log\hat{p}\left(\lambda \right)+\left(1-Y\right)\log\left(1-\hat{p}\left(\lambda \right)\right)\right].$$

Taking expectation with respect to the true distribution of *Y* gives

$$L\left(\lambda \right)=E\left[l\left(\hat{p}\left(\lambda \right),Y\right)\right]=-\left[p^{*}\log\left(1-e^{-\lambda }\right)+\left(1-p^{*}\right)\log\left(e^{-\lambda }\right)\right].$$

Since $\log\left(1-\hat{p}\left(\lambda \right)\right)=\log\left(e^{-\lambda }\right)=-\lambda$, this simplifies to

$$L\left(\lambda \right)=-p^{*}\log\left(1-e^{-\lambda }\right)+\left(1-p^{*}\right)\lambda .$$

Differentiating with respect to λ yields

$$L^{\prime }\left(\lambda \right)=-p^{*}\frac{e^{-\lambda }}{1-e^{-\lambda }}+\left(1-p^{*}\right).$$

Setting $L^{\prime }\left(\lambda \right)=0$ gives

$$\left(1-p^{*}\right)=p^{*}\frac{e^{-\lambda }}{1-e^{-\lambda }}.$$

Solving for λ yields

$$e^{-\lambda }=1-p^{*}.$$

Hence

$$\lambda =-\log(1-p^{*}).$$

Because $1-p^{*}=P\left(G=0\right)$, we obtain

$$\lambda =-\log P\left(G=0\right)=xG^{+}.$$

Therefore, the expected log-loss is minimized at $\lambda =xG^{+}$.

### Appendix D.8. Proof of Proposition A2

For a Bernoulli event with true probability $p^{*}$ and predicted probability $\hat{p}$, the expected log-loss equals the cross-entropy

$$L=-\left[p^{*}\log\hat{p}+\left(1-p^{*}\right)\log\left(1-\hat{p}\right)\right].$$

This quantity admits the decomposition

$$L=H\left(p^{*}\right)+D_{\mathrm{KL}}\;\left(\mathrm{Bern}\left(p^{*}\right)∥\mathrm{Bern}\left(\hat{p}\right)\right),$$

which separates the intrinsic entropy of the data from the divergence between the true and predicted distributions [6].

Substituting $\hat{p}\left(\lambda \right)=1-e^{-\lambda }$ shows that minimizing the expected log-loss is equivalent to minimizing the KL divergence between the true Bernoulli distribution and the Poisson-induced predictor. The divergence is minimized when

$$\hat{p}\left(\lambda \right)=p^{*}.$$

Solving $1-e^{-\lambda }=p^{*}$ yields

$$\lambda =-\log\left(1-p^{*}\right)=xG^{+}.$$

## Appendix E. Exploratory Benchmark Against Alternative Binary-Event Predictors

As an additional exploratory benchmark, we compared the proposed Poisson-based predictors with simple logistic-regression models for the binary event G≥1. Evaluation was performed using leave-one-match-out cross-validation at the match level so that both team–match observations from the same match were held out together. Table A2 reports the resulting log-loss and Brier scores.

**Table A2:** Exploratory comparison of alternative predictors for the event G≥1 using leave-one-match-out cross-validation at the match level. In the logistics specifications, shot count refers to the number of shots the team takes in a given match. Bold values indicate the better-performing model for each metric.

Model	Logloss ↓	Brier ↓	Δ Logloss
Pois$\left(xG^{+}\right)$	**0.485**	**0.161**	0.000
Logistic$\left(xG^{+}+\mathrm{shot}\;\mathrm{count}\right)$	0.486	0.161	0.001
Logistic(xG+shotcount)	0.487	0.161	0.002
Logistic$\left(xG^{+}\right)$	0.489	0.162	0.004
Logistic(xG)	0.497	0.164	0.012
Pois(xG)	0.504	0.167	0.019

These results show that, in the analyzed dataset, the event-specific Poisson mapping underlying $xG^{+}$ yields the most favorable predictive performance among the compared specifications. At the same time, the best logistic alternatives are very close, suggesting that part of the same event-level information can also be captured by simple, data-driven binary predictors. Accordingly, this comparison should be interpreted as a supplementary robustness check rather than as a definitive predictive ranking.

As a robustness check, we also repeated the binary-event benchmark separately for each league. Table A3 shows league-specific log-loss and Brier scores for the two Poisson-based predictors and the best-performing logistic alternative.

**Table A3:** League-specific robustness check for alternative predictors of the event G≥1. Results are based on leave-one-match-out cross-validation at the match level. The last column reports the difference in log-loss relative to the best-performing model within each league. In the logistic specifications, shot count refers to the number of shots the team takes in a given match. Bold values indicate the best-performing model within each league for each metric.

League	Model	Logloss ↓	Brier ↓	Δ Logloss
	Pois$\left(xG^{+}\right)$	**0.4336**	**0.1425**	0.0000
1. Bundesliga	Logistic$\left(xG^{+}+\mathrm{shot}\;\mathrm{count}\right)$	0.4338	0.1431	0.0002
	Pois(xG)	0.4609	0.1496	0.0273
	Pois$\left(xG^{+}\right)$	**0.4898**	**0.1641**	0.0000
La Liga	Logistic$\left(xG^{+}+\mathrm{shot}\;\mathrm{count}\right)$	0.4924	0.1642	0.0026
	Pois(xG)	0.5074	0.1693	0.0176
	Pois$\left(xG^{+}\right)$	**0.5138**	**0.1714**	0.0000
Premier League	Logistic$\left(xG^{+}+\mathrm{shot}\;\mathrm{count}\right)$	0.5186	0.1728	0.0048
	Pois(xG)	0.5254	0.1752	0.0116
	Pois$\left(xG^{+}\right)$	**0.4917**	0.1624	0.0000
Serie A	Logistic(xG+shotcount)	0.4932	**0.1622**	0.0015
	Pois(xG)	0.5135	0.1695	0.0218

Table A3 indicates that the pooled results are qualitatively stable across leagues. In each competition, Pois$\left(xG^{+}\right)$ improves on Pois(xG), while the best logistic alternative remains very close to the $xG^{+}$-based predictor. This suggests that the main empirical pattern is not driven by a single league.

## References

[1] Rathke A. (2017). An Examination of Expected Goals and Shot Efficiency in Soccer. J. Hum. Sport Exerc..

[2] Spearman W. Beyond Expected Goals. Proceedings of the MIT Sloan Sports Analytics Conference.

[3] Anzer G., Bauer P. (2021). A Goal Scoring Probability Model for Shots Based on Synchronized Positional and Event Data in Football (Soccer). Front. Sports Act. Living.

[4] Dixon M.J., Coles S.G. (1997). Modelling Association Football Scores and Inefficiencies in the Football Betting Market. J. R. Stat. Soc. Ser. C Appl. Stat..

[5] Baio G., Blangiardo M. (2010). Bayesian Hierarchical Model for the Prediction of Football Results. J. Appl. Stat..

[6] Cover T.M., Thomas J.A. (2006). Elements of Information Theory.

[7] Shannon C.E. (1948). A Mathematical Theory of Communication. Bell Syst. Tech. J..

[8] Lopes A.M., Tenreiro Machado J.A. (2019). Entropy Analysis of Soccer Dynamics. Entropy.

[9] Bandara I., Shelyag S., Rajasegarar S., Dwyer D., Kim E.J., Angelova M. (2026). Maximizing Ball Movement Unpredictability in Association Football: A Rényi Entropy-Based Approach to Optimizing Event Distribution Randomness. PLoS ONE.

[10] Bandara I., Shelyag S., Rajasegarar S., Dwyer D., Kim E.J., Angelova M. (2024). Winning with Chaos in Association Football: Spatiotemporal Event Distribution Randomness Metric for Team Performance Evaluation. IEEE Access.

[11] Cheng Y.S., Chang A.Y.C., Doya K. (2025). Information-Theoretical Analysis of Team Dynamics in Football Matches. Entropy.

[12] Cavus M., Biecek P. Explainable Expected Goal Models for Performance Analysis in Football Analytics. Proceedings of the 2022 IEEE 9th International Conference on Data Science and Advanced Analytics (DSAA).

[13] Mead J., O’Hare A., McMenemy P. (2023). Expected Goals in Football: Improving Model Performance and Demonstrating Value. PLoS ONE.

[14] Hong Y. (2013). On Computing the Distribution Function for the Poisson Binomial Distribution. Comput. Stat. Data Anal..

[15] Simpson E.H. (1949). Measurement of Diversity. Nature.

[16] Brier G.W. (1950). Verification of Forecasts Expressed in Terms of Probability. Mon. Weather Rev..

[17] Gneiting T., Raftery A.E. (2007). Strictly Proper Scoring Rules, Prediction, and Estimation. J. Am. Stat. Assoc..

[18] Heinrich-Mertsching C., Thorarinsdottir T.L., Guttorp P., Schneider M. (2024). Validation of Point Process Predictions with Proper Scoring Rules. Scand. J. Stat..

[19] Waghmare K., Ziegel J. (2026). Proper Scoring Rules for Estimation and Forecast Evaluation. Annu. Rev. Stat. Appl..

[20] Niculescu-Mizil A., Caruana R. Predicting Good Probabilities with Supervised Learning. Proceedings of the 22nd International Conference on Machine Learning.

[21] Van Calster B., McLernon D.J., van Smeden M., Wynants L., Steyerberg E.W. (2019). Calibration: The Achilles Heel of Predictive Analytics. BMC Med..

[22] Marshall A.W., Olkin I., Arnold B.C. (2011). Inequalities: Theory of Majorization and Its Applications.

